# Ileal leiomyoma disguised as intussusception: Imaging pitfalls in a 28-year-old female

**DOI:** 10.18632/oncoscience.656

**Published:** 2026-04-15

**Authors:** Reshmi Sultana, AM Adarsha, Tushar M. Parmeshwar, Akula Nynasindhu, Abhimanyu Sharma

**Affiliations:** ^1^Department of General Surgery, All India Institute of Medical Sciences, Bibinagar, Telangana 508126, India; ^2^Department of Pathology, All India Institute of Medical Sciences, Bibinagar, Telangana 508126, India

**Keywords:** leiomyoma, intussusception, bowel, adnexal tumour

## Abstract

Leiomyomas are benign mesenchymal tumors that commonly occur in uterus and rarely in gastrointestinal tract with esophagus being the most frequent site followed by stomach and colon. Duodenal leiomyoma is rarest of all with ileal leiomyoma being the second rarest. This report discusses a case of a 28-year-old female with an enlarging lower abdominal mass post-hysterectomy, initially misinterpreted as an adnexal tumor based on imaging. Various diagnostic modalities, including ultrasound, CECT, and MRI, provided conflicting interpretations, complicating preoperative assessment. Exploratory laparotomy confirmed an intraluminal ileal leiomyoma, highlighting the importance of integrated imaging and histopathological evaluation for accurate diagnosis. Genetic studies indicate associations with syndromic conditions like NF1 and Reed syndrome, emphasizing the need for vigilant surveillance and potential molecular-targeted therapies. While traditionally managed surgically, emerging research suggests epigenetic modifications may play a role in tumor behaviour and therapeutic advancements.

## INTRODUCTION

Mesenchymal tumors, encompassing benign, intermediate, and malignant entities, are a diverse group of neoplasms with a global incidence of 30–50 cases per million person-years [1]. Leiomyomas, benign mesenchymal tumors commonly found in the uterus, rarely occur in extrauterine sites. Small bowel leiomyomas account for 1% of smooth muscle tumors, primarily affecting the jejunum (44%), ileum (37%), and duodenum (19%) [2]. Although often asymptomatic, affected individuals may present with abdominal pain, bleeding, or acute intestinal obstruction, sometimes involving intussusception [3].

## CASE REPORT

A 28-year-old female presented with a progressively enlarging lower abdominal mass associated with intermittent colicky pain over two months. She reported a weight loss of 4 kg during this period. Previously diagnosed with uterine fibroids, she had undergone laparoscopic hysterectomy; however, her symptoms persisted.

**Figure 1:**
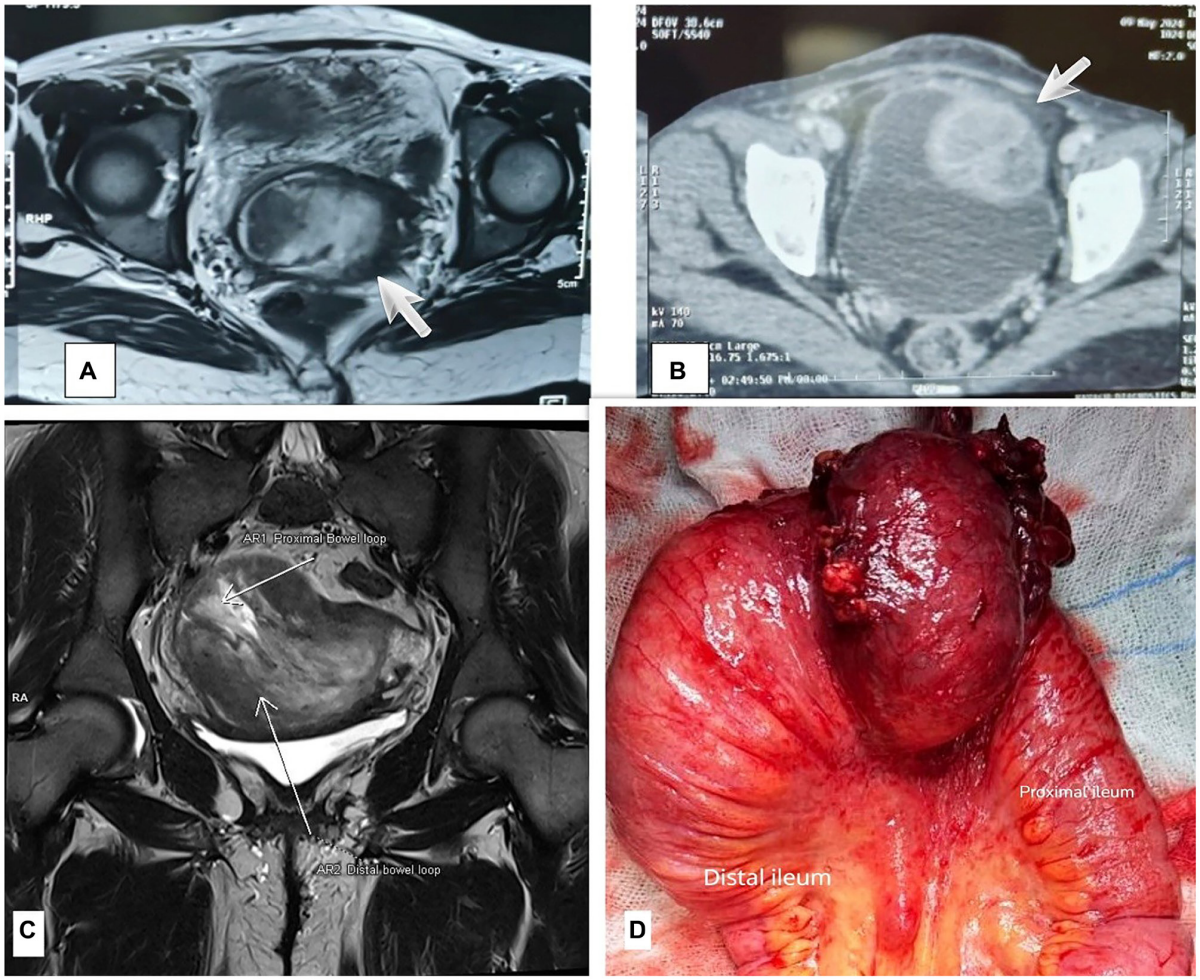
From right to left. (**A**) Coronal T2 sequence heterogenous hyperintense mass seen within the small bowel. (**B**) CECT abdomen and pelvis coronal and sagittal section showing large well-defined heterogenous density. (**C**) Coronal T2 weighted image showing intussusception with markings of proximal and distal bowel loop. (**D**) Intra-op image of lesion in ileum.

Examination revealed a non-tender, firm swelling of approximately 5 × 4 cm in the right iliac fossa with limited mobility. Investigations though had inconclusive findings:

USG showed hyperechoic lesion possibly in right adnexa measuring 5.3 × 4.5 cm suggestive of right ovarian dermoid. (Figure 1).

CECT abdomen and pelvis have added to the diagnostic dilemma with the finding of a large, well-defined heterogeneous density measuring 7 × 10 × 8 cm, suggesting a postoperative hematoma with infection. This interpretation is likely influenced by post-hysterectomy pelvic changes and inflammation, which distort anatomical clarity and obscure imaging. (Figure 1).

MRI pelvis suggested a large, T1 and T2 hyperintense mass of 8 × 5.5 cm ileo-ileal intussusception mass. (Figure 1).

**Figure 2:**
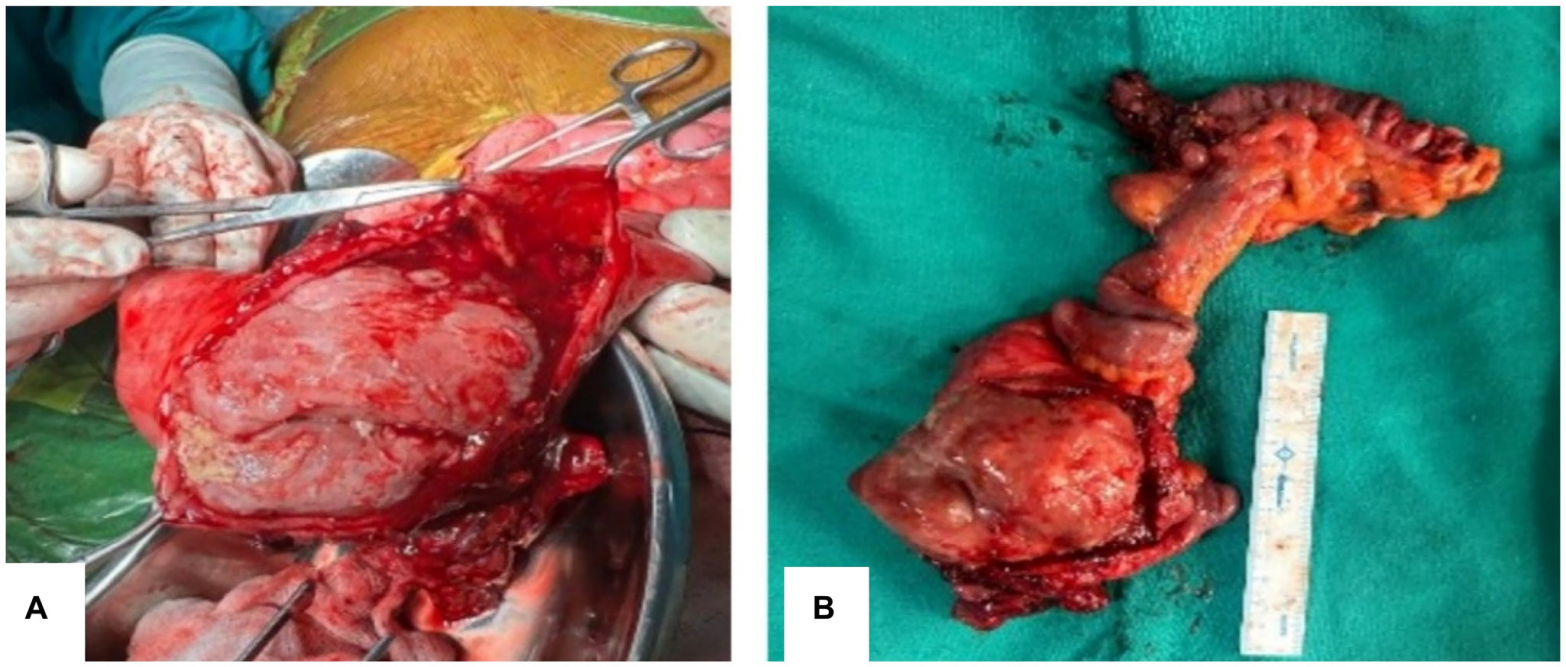
(**A**) Cut-section of the tumour occupying almost entire circumference. (**B**) Specimen showing intraluminal fleshy tumour arising from wall of ileum.

**Figure 3:**
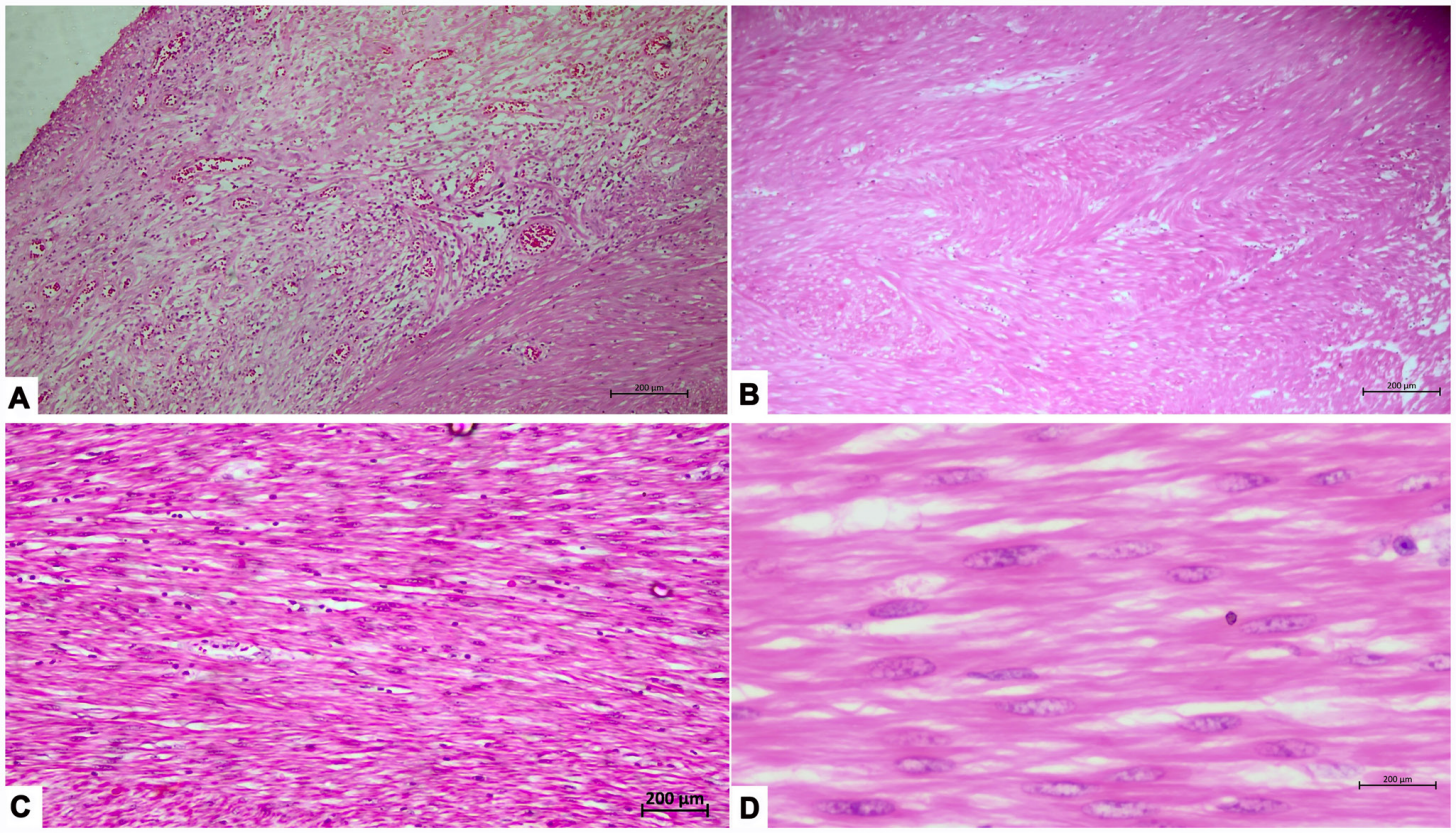
(**A**) Low power view showing ulcerated intestinal mucosa and underlying tumor (×50 magnification, Hematoxylin and eosin). (**B**) Low power view showing tumor arranged in sheets and fascicles (×50 magnification, Hematoxylin and eosin). (**C**) Tumor arranged in sheets and fascicles consisting of spindle shaped cells with cigar shaped nucleus (×100 magnification, Hematoxylin and eosin). (**D**) Higher magnification shows a tumor consisting of spindle shaped cells with cigar shaped nucleus and eosinophilic cytoplasm (×400 magnification, Hematoxylin and eosin).

An exploratory laparotomy revealed a 10 × 8 × 5 cm intraluminal ileal mass located 15 cm proximal to the ileocecal junction. The mass was resected with adequate margins, followed by ileo-ascending colon anastomosis. (Figure 2). On cut section, the tumor exhibited intraluminal growth almost obscuring entire circumference. The target sign, typically formed by concentric rings of soft tissue and fluid, can misleadingly appear in such cases, mimicking intussusception. Histopathology reported a spindle-cell tumor with minimal atypia and a low mitotic index (<1/10 hpf), indicative of a small bowel leiomyoma. (Figure 3). Immunohistochemistry (IHC) confirmed the diagnosis with positive desmin and S100 markers and negative CD117 and CD34 markers.

## DISCUSSION

Leiomyomas are benign mesenchymal tumors of smooth muscle origin, accounting for one-third of gastrointestinal mesenchymal neoplasms [1]. Gastrointestinal tract leiomyomas, though rare, represent the most prevalent type of benign nonepithelial tumours found in the small intestine as first described by Virchow in 1854 [4]. These tumors arise from monoclonal smooth muscle cells embedded within extracellular matrix and are encapsulated by a pseudo capsule of areolar tissue [5].

Symptoms such as obstruction, bleeding, intussusception, or palpable abdominal mass manifest in 44–50% of cases, while smaller lesions are often incidentally detected during surgical procedures or imaging studies [6].

Although rare, small intestinal leiomyomas can be associated with genetic syndromes. Reed syndrome (HLRCC) primarily causes cutaneous and uterine leiomyomas but may occasionally affect the small bowel, with a high risk of aggressive renal carcinoma. NF1 can present with multiple small bowel leiomyomas. Syndromes associated with overlapping differentials are Familial GIST syndrome, driven by KIT and PDGFRA mutations, predisposes individuals to multiple GISTs, while Carney triad includes GISTs, pulmonary chondromas, and paragangliomas. MEN-1 is linked to neuroendocrine tumors in the bowel, whereas Peutz-Jeghers syndrome is characterized by hamartomatous polyps.

Akman et al reported a case of a 69-year-old postmenopausal woman with a right iliac fossa mass, initially misinterpreted as an adnexal tumor on imaging but later confirmed as an ileal leiomyoma through IHC, mirroring our case. The close anatomical proximity of the terminal ileum to the adnexa, coupled with the difficulty in distinguishing bowel tumors radiologically, complicates diagnosis and underscores the crucial role of tissue-based analysis [3].

Histologically, leiomyomas consist of spindled cells with eosinophilic cytoplasm and blunt-ended nuclei, lacking necrosis and mitotic activity—critical factors in assessing malignant potential [7, 8]. While most remain benign, rare instances of malignant transformation into leiomyosarcoma have been reported, though the exact frequency remains unclear. Tumors with increased mitotic activity, necrosis, or rapid growth require vigilant surveillance, as genetic mutations like TP53 alterations may elevate malignancy risk. IHC markers, including desmin, smooth muscle actin (SMA), h-caldesmon, and calponin, help differentiate leiomyomas from gastrointestinal stromal tumors (GISTs), which are often malignant and express KIT (CD117) and DOG1. GISTs, often malignant, benefit from tyrosine kinase inhibitors.

Surgical resection is the preferred treatment for gastrointestinal leiomyomas, with approach depending on tumor size, location, and surrounding structures. Open surgery provides better visualization, but laparoscopic techniques are increasingly favoured for their faster recovery and fewer complications. Ensuring adequate resection margins is crucial to prevent recurrence, and intraoperative frozen section analysis helps assess malignancy risk. Recent research suggests that epigenetic modifications, including DNA methylation and histone acetylation, contribute to tumor progression by influencing gene expression and cellular behavior. Understanding these molecular mechanisms may help identify novel therapeutic targets, expanding treatment options beyond surgical intervention [9].

## CONCLUSIONS

Leiomyoma of ileum, though benign, present diagnostic challenges due to imaging similarities with other abdominal pathologies. Accurate differentiation requires integrated imaging, histopathology, and immunohistochemistry. Surgical resection is key, but optimal approach, adequate margin to prevent recurrence, and long-term surveillance remain crucial. Emerging genetic insights offer potential for advanced diagnostics and targeted therapies, emphasizing a multidisciplinary approach for optimal patient care.
